# Temporospatial Flavonoids Metabolism Variation in *Ginkgo biloba* Leaves

**DOI:** 10.3389/fgene.2020.589326

**Published:** 2020-11-27

**Authors:** Ying Guo, Tongli Wang, Fang-Fang Fu, Yousry A. El-Kassaby, Guibin Wang

**Affiliations:** ^1^Co-Innovation Centre for Sustainable Forestry in Southern China, Nanjing Forestry University, Nanjing, China; ^2^College of Forestry, Nanjing Forestry University, Nanjing, China; ^3^Department of Forest & Conservation Sciences, Faculty of Forestry, The University of British Columbia, Vancouver, BC, Canada

**Keywords:** *Ginkgo biloba*, flavonoids biosynthesis, leaf development, transcriptome dynamics, temporospatial variation

## Abstract

Ginkgo (*Ginkgo biloba* L.) is a high-value medicinal tree species characterized by its flavonoids beneficial effects that are abundant in leaves. We performed a temporospatial comprehensive transcriptome and metabolome dynamics analyses of clonally propagated Ginkgo plants at four developmental stages (time: May to August) across three different environments (space) to unravel leaves flavonoids biosynthesis variation. Principal component analysis revealed clear gene expression separation across samples from different environments and leaf-developmental stages. We found that flavonoid-related metabolism was more active in the early stage of leaf development, and the content of total flavonoid glycosides and the expression of some genes in flavonoid biosynthesis pathway peaked in May. We also constructed a co-expression regulation network and identified eight *GbMYB*s and combining with other TF genes (3 *GbERF*s, 1 *GbbHLH*, and 1 *GbTrihelix*) positively regulated the expression of multiple structural genes in the flavonoid biosynthesis pathway. We found that part of these *GbTF*s (Gb_11316, Gb_32143, and Gb_00128) expressions was negatively correlated with mean minimum temperature and mean relative humidity, while positively correlated with sunshine duration. This study increased our understanding of the molecular mechanisms of flavonoids biosynthesis in Ginkgo leaves and provided insight into the proper production and management of Ginkgo commercial plantations.

## Introduction

Ginkgo (*Ginkgo biloba* L.) leaves contain a variety of medicinal compounds, which have been used in healthcare and food industries. Flavonoids are the major bioactive ingredients in Ginkgo leaves, including flavonols, flavones, and anthocyanins (Meng et al., 2019). These molecules have been reported to have beneficial effects in preventing metabolic syndrome at different levels such as early stage Alzheimer’s and cardiovascular diseases (Tian et al., 2017; Gruenwald et al., 2020). Flavonoids also act as growth regulators controlling single organ and whole plant development (Agati et al., 2012). Therefore, it is essential to understand the molecular mechanisms of flavonoids accumulation during Ginkgo leaves development to ultimately improve the production and management of Ginkgo plantations.

Recently, considerable efforts have been dedicated to improving Ginkgo leaves flavonoids for commercial production. Studies showed that several agronomic measures could increase the flavonoids content, such as alternative partial root-zone irrigation (Wang et al., 2016), fertilization (Guo et al., 2016), and foliar fertilization (Wu et al., 2020). Treatments with salicylic acid, UV-B, and NaCl, all have shown a positive effect on increasing Ginkgo leaves flavonoids content (Ni et al., 2017, 2018; Zhao et al., 2020). More importantly, additional efforts have been directed at the molecular level to achieve the same objective. For example, transcriptome libraries have been constructed for various Ginkgo tissues (Ye et al., 2019) and leaves with different flavonoid contents (Wu et al., 2018), for improving the understanding of flavonoid biosynthesis. Another strategy for improving Ginkgo leaves metabolites yield is through genetic engineering; however, detailed information on gene expression profiling and transcriptional dynamics that regulate flavonoids accumulation is scarce.

Leaves undergo a series of developmental and physiological changes during their lifespans, involving complex, but highly regulated molecular processes to maximize fitness in a given ecological setting (Leopold, 1961; Fenner, 1998). It was found that leaves from the same Ginkgo tree could exhibit differences in flavonoids content at different developmental stages (young vs. mature leaves) (Guo et al., 2020). Additionally, Ginkgo leaves from plants growing at different elevations (different environments), and the same growing period also displayed substantial differences in their flavonoids accumulation (Zou et al., 2019). However, the current understanding of flavonoids accumulation regulation mechanism, which varies according to the development stage and geographical distribution, is limited. Transcriptomes can provide information regarding gene expression and regulation at specific developmental stages or under specific physiological conditions (Sato et al., 2011). Furthermore, integration of different-omics data, such as metabolome, will help elucidate the complex mechanism controlling flavonoid biosynthesis (Weckwerth, 2008).

Here, we conducted comprehensive temporospatial transcriptome and metabolome dynamics analyses of clonally propagated Ginkgo plants at four developmental stages (May to August) across three different environments (test-sites) to unravel leaves flavonoids biosynthesis spatial-temporal variation. The study-specific objectives are to: (1) quantify the transcriptional responses to spatial (environmental cues) and temporal (development stages) conditions; (2) explore the association between flavonoids accumulation and expression of flavonoid related structural genes; and (3) elucidate the regulatory network involved in gene expression associated with flavonoids biosynthesis. The broader aim of this work is intended to improve our understanding of the transcriptional dynamics that regulate flavonoids accumulation at the molecular level and provide insightful information for enhancing flavonoids content of Ginkgo leaves for the proper production and management of Ginkgo commercial plantations.

## Materials and Methods

### Plant Materials and Sample Collection

Generally, the optimum age of flavonoids production in Ginkgo leaf-harvest plantations is trees under 5-year-old (Zou et al., 2019), thus older trees are considered suboptimum. Therefore, in the present study we utilized leaf samples collected from 2-year-old clonally propagated (grafted) Ginkgo trees. Trees are spatially replicated over three test sites (i.e., different environments) (Table 1). These sites are: (1) Yi Ning (YN), located in northwestern China (lat.: 43.41°N, long.: 81.11°E), characterized by a typical mid-temperate continental semi-arid climate; (2) Pi Zhou (PZ), located in central China (lat.: 34.21°N, long.: 117.58°E) characterized by a warm temperate monsoon climate; and (3) Qu Jing (QJ), located in southern China (lat.: 25.52°N, long.: 103.58°E), characterized by a subtropical plateau monsoon climate. In each site, the experiment is planted as a complete randomized block design with three blocks (replicates), each harboring 20 Ginkgo clones.

**TABLE 1:** Geographical distribution and climate factors [mean annual temperature (MAT), mean annual precipitation (MAP), and mean annual sunshine duration (MASD)] of the studied three test sites [Yi Ning (YN), Pi Zhou (PZ), and Qu Jing (QJ)].

Site	Latitude (°N)	Longitude (°E)	Altitude (m)	MAT (°C)	MAP (mm)	MASD (h)
YN	43.41	81.11	820	5.2	331	7.1
PZ	34.21	117.58	44	14.5	845	5.9
QJ	25.52	103.58	2,160	14.1	1,067	6.5

Samples were conducted between leaf expansion (May, after majority of leaves expansion) and leaf “commercial” ripening (August, before autumnal senescence). During this biological window, Ginkgo leaves are at their substance’s peak activity and are easy to harvest and store (Ellnain-Wojtaszek et al., 2002). Leaves were collected on a clear day in the middle of each month (May to August) to represent four temporal leaf developmental stages. A single clone was randomly selected across the three blocks (i.e., 3 biological replications) and the collected leaf samples provided the material for the metabolomics and transcriptomics analyses. Each sampled tree was represented by three crown positions (top, middle, and bottom), each provided a single complete and healthy leaf. In total, 36 samples (4 development stages × 3 environments × 3 biological replicates) were used for the metabolomics and transcriptomics analyses. Collected leaves were immediately preserved in liquid nitrogen, freeze-dried, and kept at −80°C until further use. To measure the temporal variation in total flavonoid glycosides (TFG) content, a monthly time series sampling was conducted (at mid-month between May and August) on the PZ site and nine leaves were randomly collected from the 20 Ginkgo clones planted in each block. Additionally, to measure the spatial changes of TFG content across environments, nine leaves were randomly collected from the same 20 Ginkgo clones from the three blocks and the same sampling scheme was conducted across the three sites (sampling was conducted in mid-August). Leaves were oven-dried (70°C, 48 h), crushed, sieved through a 100-mesh sieve, and vacuum packed. All experiments were performed with three biological replicates.

### Total Flavonoid Glycosides Measurement

Ginkgo leaves flavonoids were extracted following the Pharmacopoeia of the People’s Republic of China (PPRC) procedures (Commision, 2010), and flavonoid glycosides content were determined by high-performance liquid chromatography (HPLC). In brief, approximately 0.5 g of oven-dried leaf powder per sample was immersed in petroleum ether and refluxed at 70°C for 2 h to remove impurities. Samples were then soaked in methanol and each sample’s extract was evaporated on a rotary evaporator after refluxed at 70°C for 4 h. Subsequently, the pellet was washed with 25 mL of a 25% methanol-HCl (4:1, v/v) mixture and the eluent was collected and refluxed for 30 min. After cooling to room temperature, the eluent was brought to 50 mL with methanol, then used for determination by HPLC. HPLC (Waters 1525, United States) conditions were set as follows: the mobile phase was methanol and 0.4% H_3_PO_4_ solution (56:44, v/v) at 1.0 mL min^–1^; the column temperature was 30°C; the detection was performed at 360 nm. Quercetin, kaempferol, and isorhamnetin were selected as standard substances following the supplier’s specifications (Yuanye Biological Co., Shanghai, China).

Total flavonoid glycosides content = (quercetin + kaempferol + isorhamnetin) × 2.51 (Commision, 2010). Means and standard errors for each sample were calculated. Differences among samples were determined using one-way ANOVA and significant differences were detected (defined as *P* < 0.05) using the least significant difference (LSD) test.

### Metabolomics Analysis

The supernatant extraction for each sample was performed as previously described (Guo et al., 2020). In summary, about 50 mg freeze-dried sample was put into an EP tube after grinding. After the addition of 1 mL of extract solvent (acetonitrile-methanol-water, 2:2:1, containing 0.1 mg L^–1^ lidocaine as an internal standard), the samples were swirled for 30 s, homogenized at 45 Hz for 4 min, and sonicated for 5 min in an ice water bath. The homogenate and sonicate circle was repeated three times, followed by incubation at −20°C for 1 h and centrifugation at 12,000 rpm and 4°C for 15 min. The resulting supernatants were transferred to LC-MS vials and stored at −80°C for later use. LC-MS/MS analyses were performed using an UHPLC system (1290, Agilent Technologies) with a UPLC HSS T3 column coupled to Q Exactive (Orbitrap MS, Thermo). The mobile phase A was 0.1% formic acid in water for positive, and 5 mmol/L ammonium acetate in water for negative, and the mobile phase B was acetonitrile. The elution gradient was set as follows: 0 min, 1% B; 1 min, 1% B; 8 min, 99% B; 10 min, 99% B; 10.1 min, 1% B; 12 min, 1% B [see Supplementary Figure S1 for a UHPLC chromatogram of standards and samples from Yi Ning (YN) site at different sampling stages]. MS raw data files were converted to the mzML format using ProteoWizard, and processed by R package XCMS. OSI-SMMS (version 1.0, Dalian Chem Data Solution Information Technology Co. Ltd.) was used for peak annotation after data processing with an in-house MS/MS database. The metabolites were mapped to the Kyoto Encyclopedia of Genes and Genomics (KEGG) metabolic pathways to identify the substances in the related pathways of flavonoid biosynthesis (ko 00941- ko 00944).

### Transcriptomics Analysis

Total RNA extraction, library preparation, and sequencing for each sample (36 libraries: four developmental stages in three different environments with three biological replicas) were performed as previously described (Guo et al., 2020). Total RNA was extracted from the freeze-dried samples using Trizol reagent kit (Invitrogen, Carlsbad, CA, United States) according to the manufacturer’s protocol. RNA quality was assessed on an Agilent 2100 Bioanalyzer (Agilent Technologies, Palo Alto, CA, United States) and checked using RNase free agarose gel electrophoresis. Then the enriched mRNA was fragmented into short fragments using fragmentation buffer and reverse transcribed into cDNA with random primers. Second-strand cDNA were synthesized by DNA polymerase I, RNase H, dNTP and buffer. Then the cDNA fragments were purified with QiaQuick PCR extraction kit (Qiagen, Venlo, Netherlands), end repaired, poly (A) added, and ligated to Illumina sequencing adapters. The ligation products were size selected by agarose gel electrophoresis, PCR amplified, and sequenced using Illumina HiSeq2500. The Ginkgo Illumina raw sequencing data were submitted to the NCBI BioProject database under project number PRJNA657336.

An index of the reference genome was built, and paired-end clean reads were mapped to the Ginkgo’s reference genome^1^ using Hisat2. The mapped outputs were processed via StringTie software to obtain FPKM (fragment per kilobase of transcript per million mapped reads) for all the Ginkgo genes in each sample. Based on gene expression, principal component analysis (PCA) and hierarchical clustering analysis were performed with R packages, *gmodels* and *pheatmap*^2^, which were also used to reveal the relationship among samples. The FPKM data were directly used to estimate the differential expression of genes (DEGs) between samples. FDR < 0.05 and | log2FC| > 1 were used as thresholds to identify significant DEGs. The Short Time-series Expression Miner (STEM) software was used to obtain the temporal expression profile of DEGs. Subsequently, DEGs in enriched clustered profiles were used for KEGG pathway enrichment analysis (*Q* value ≤ 0.05) to assess metabolic pathways and related gene functions.

Weighted gene co-expression network analysis (WGCNA) was performed in the R environment. After filtering with the R package DCGL, a total of 23,182 genes (FPKM > 0) were reserved for subsequent analysis. The adjacency matrix between different genes was constructed with a threshold power of 10. A dynamic tree cut procedure (merge cut height = 0.70, min module size = 50) in R package WGCNA was used to identify similar modules in the hierarchical tree. The expression profile of module genes in each sample was displayed by module eigengene, which was defined as the first principal component of a given module. The Pearson correlations between the eigengenes of each module and the abundance of flavonoids were plotted by R package ggplot2. Subsequently, we identified the encoding transcription factor (TF) genes and the structural genes in the biosynthesis pathways of related flavonoids from the target modules. The gene regulatory networks were generated by Cytoscape software (Version 3.7.1).

The promoter region of TF genes was analyzed for presence of cis-acting regulatory elements by PlantCARE^3^ and visualized by TBtools software. Additionally, to explore the regulatory effect of environmental factors on the expression of TF genes, Pearson’s product-moment correlation analysis was conducted between TF genes expression and environmental factors during development (daily meteorological data for each area from May to August 2019^4^).

### Quantitative Real-Time PCR (qRT-PCR) Analysis

Ten genes involved in flavonoid biosynthesis were randomly chosen for validation by qRT-PCR. According to the manufacturer’s instructions, cDNA was obtained using MonScript RTIII All-in-One Mix with dsDNase kits (Monad, China) and qRT-PCR analysis was carried out using an Applied Biosystems^TM^ 7500 Real-Time PCR Systems (Monad, China). Primers used were designed in Primer Premier 5 (United States), and the primer sequences are provided in Supplementary Table S2. Glyceraldehyde 3- phosphate dehydrogenase (GAPDH, GenBank Accession No. L26924) gene was used as an internal standard. The relative transcript abundance was calculated using the 2^–ΔΔC_T^ method (Livak and Schmittgen, 2001). August samples from YN, PZ, and QJ sites were used for qRT-PCR analysis. As designed, each sample included three biological replicates and three independent technical repetitions.

### Statistical Analysis

All statistical analyses were conducted in R environment (R Core Team, 2019). Differences among TFGs content were determined using one-way analysis of variance (ANOVA) and significant differences were calculated using the least significant difference (LSD) test (defined as *P* < 0.05). The complex relationships between gene expression profiles were intuitively displayed by a PCA plot and a cluster heat map. Relationships between expression of structural genes and abundance of flavonoids were evaluated using the Pearson’s product-moment correlation analysis (*P* < 0.05, significant correlation).

## Results

### Changes in Total Flavonoid Glycosides Content

**FIGURE 1:**
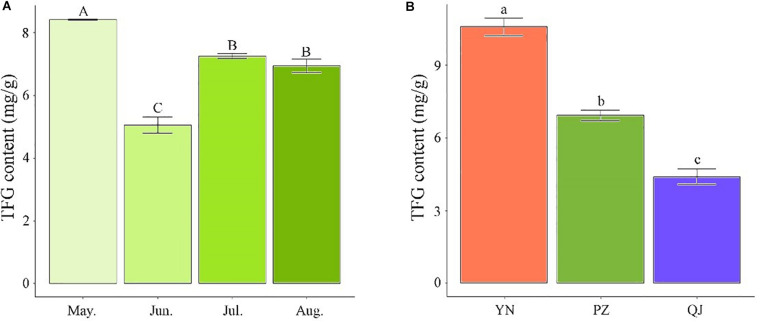
Differences in total flavonoid glycosides (TFG) content among Ginkgo leaf samples. **(A)** Temporal differences in TFG content among developmental stages in PZ site. **(B)** Spatial differences in TFG content in August-samples across the three environments. Measurements were performed in triplicates. Error bars indicate standard deviations, and different capital and small letters represent a significant difference (*P* < 0.05) between developmental stages and environments, respectively.

Temporarily, TFG content showed a declining trend with sampling time during the leaf development process. PZ site’s TFG content time-course analysis showed the highest value occurred in May, followed by a drastic drop in June (*P* < 0.05) and a significant recovery in July (Figure 1A). Compared to May samples, the TFG content of June, July, and August samples decreased by 66.40, 15.98, and 21.50%, respectively (Figure 1A). Spatially, apparent differences in TFG content were observed across the three growing environments. Compared to August’s PZ and QJ samples, the TFG content of YN was larger by 52.67 and 140.45%, respectively (Figure 1B).

### Changes in Gene Expression Profile

**FIGURE 2:**
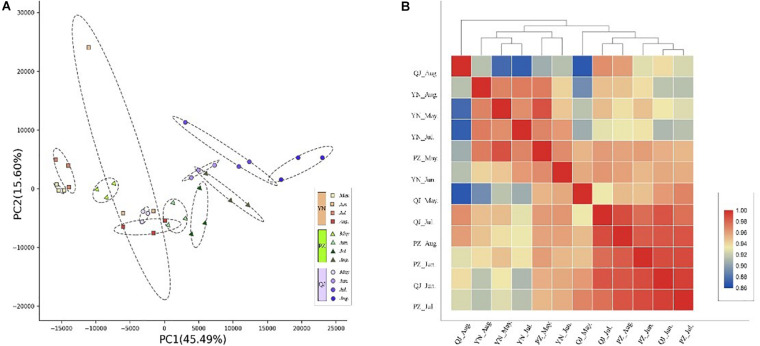
Temporospatial differences in gene expression profile. **(A)** Principal component analysis (PCA) plot showing clustering of leaf transcriptomes at four developmental stages (temporal) under three different environments (spatial). Red squares, green triangles, and blue circles represent samples from YN, PZ, and QJ sites, respectively. The change in color from light to dark represents the four leaf development stages (May to August). **(B)** Pearson product-moment correlation coefficients and clusters of the RNA-seq data from leaf samples. The redder the rectangle, the stronger the correlation between the samples, whereas the bluer the rectangle, the weaker the correlation.

Through transcriptional dynamics analysis, we identified approximately 2.0 billion clean reads from the 36 cDNA libraries that were mapped to the Ginkgo genome. The mapping rates of each library ranged from 91.14 to 95.39% (Supplementary Table S1). Spatially, PCA analysis results showed clear separation on the PC biplot, accounting for 61.1% of total gene expression variance in the data set (Figure 2A). Samples were spatially separated along the PC1 axis with YN, PZ, and QJ positioned on the left, middle, and right, respectively (Figure 2A). Temporally, within each site, the four developmental stage samples tended to follow the same left-to-right trend along PC1, while this trend did not persist for PC2 (Figure 2A). In the hierarchical clustering analysis, we did not detect any evidence of clustering among samples at either the different developmental stages (temporal) or at any given environment (spatial) (Figure 2B). Interestingly, PZ samples of the earlier stage (May) exhibited a closer correlation with the YN samples, whereas the PZ samples of a later stage (August) tended to correlate with the QJ samples, suggesting that major transcriptional program differences existed among development stages within each environment.

### Differential Gene Expression During Leaf Development

**FIGURE 3:**
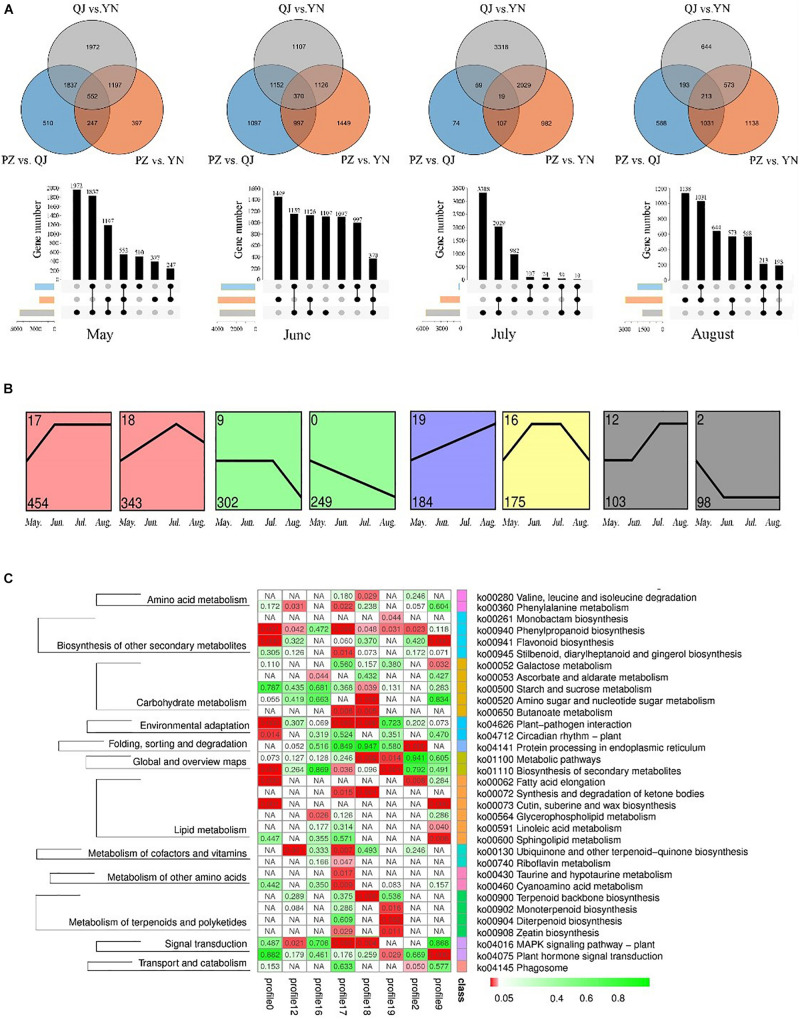
Temporospatial expression pattern of differential expressed genes (DEGs). **(A)** Venn diagrams and column charts showing DEGs between samples from different growth environments (spatial) at four development stages (temporal). The gray circle/rectangle represents the difference between samples from YN and QJ; the blue one represents the difference between samples from PZ and QJ; the orange one represents the differences between the PZ and YN samples. **(B)** Profile blocks with a colored background are significant clusters of the *P* ≤ 0.05, and the same color represents that the profiles are the same cluster. **(C)** An enriched KEGG map shows significant pathways among the genes of eight profiles. The red rectangle represents a significant enrichment pathway (*P* < 0.05).

At each leaf developmental stage, we identified different expression genes (DEGs) among samples from different environments (Figure 3A). We found more DEGs differences existed between QJ and YN samples (number of stage-specific genes varied from 644 to 3,318), while fewer DEGs differences between PZ and QJ samples (number of stage-specific genes varied from 74 to 1,097). The variable number of DEGs differences suggested that each stage of Ginkgo clones development had an independent strategy in response to their respective different environmental conditions.

To analyze the temporal expression pattern of DEGs, the 24,958 DEGs were further clustered by Short Time-series Expression Miner (STEM) software. There were 8 identifiable statistically significance (*P* < 0.05) temporal expression patterns, which were divided into 5 clusters containing a total of 1,908 DEGs (Figure 3B). The expression of DEGs contained in the 0 profile was gradually down-regulated during leaf development, while the temporal expression pattern of the 19 profile showed an opposite pattern. The KEGG pathway enrichment analysis of 1,908 DEGs revealed that 33 pathways were significantly enriched, including a large number of secondary metabolites, carbohydrate, and lipid metabolic pathways (Figure 3C). In particular, the phenylalanine and flavonoid biosynthesis pathways (ko 00940 and ko 00941) were enriched in several profiles. Therefore, these results suggested that the expression of some genes in the flavonoids-related biosynthesis pathways varied as a function of environmental factors (spatial) and developmental stages (temporal). The reliability of the RNA-seq results and the differentially expression analysis was further verified by qRT-PCR (Supplementary Figure S2).

### Identification and Screening of Gene Co-expression Modules

**FIGURE 4:**
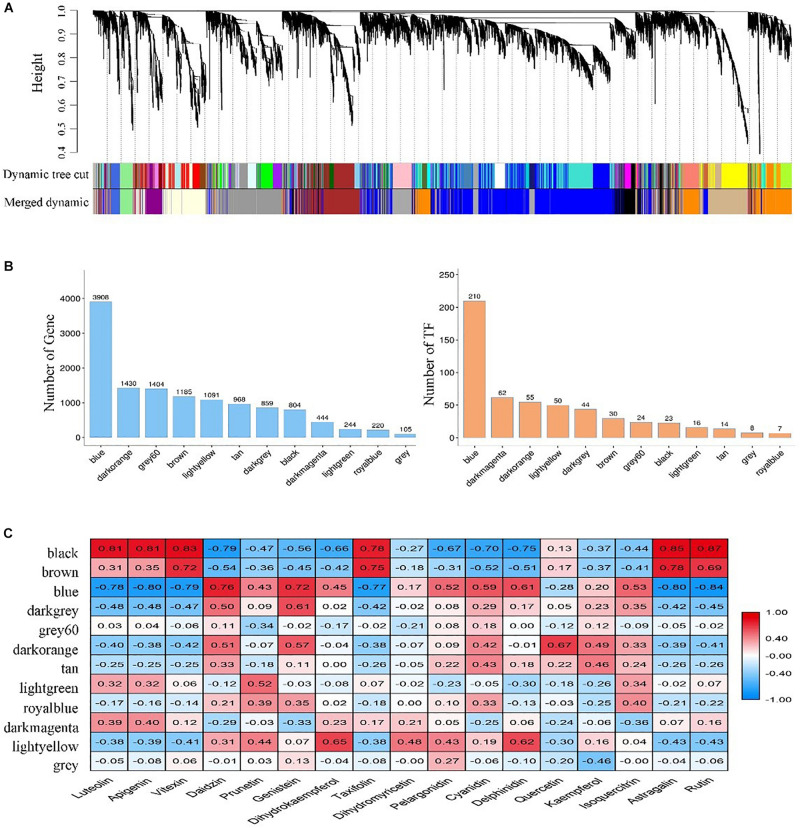
Identification and screening of gene co-expression modules. **(A)** Dendrogram showing modules identified by the weighted gene co-expression network analysis (WGCNA) and clustering dendrogram of expressed genes; **(B)** The number of genes and transcription factors (TFs) contained in each module; **(C)** Correlation coefficient between the abundance of flavonoids and module eigengenes presented with a color scale with red and blue representing positive and negative correlations, respectively.

Twelve modules were identified in a dendrogram comprising 105 – 3,908 genes, and each module harbored genes encoding the number of transcription factors (TFs) varying from 7 to 210 (Figures 4A,B). In most modules, TF-encoding genes accounted for more than 5% of the total genes, indicating that the transcriptional activity was strictly regulated. Also, the eigengene of each module was associated with the abundance of 17 flavonoids revealed by Pearson product-moment correlation coefficient analysis. Remarkably, three modules (Black, Blue, and Brown) exhibited a strong correlation (*r* > | 0.7|, *P* < 0.05) between gene expression and flavonoids accumulation (Figure 4C).

To better understand the function of genes in these three modules, we assigned the genes to KEGG terms. The top 20 enriched pathways in each module were revealed by bubble maps. The genes from Black and Blue modules were significantly enriched in pathways related to translation, folding, sorting and degradation, signal translation, amino acid metabolism, and energy metabolism (Supplementary Figures S3A,B). In these pathways, some unigenes encoding glutathione S-transferase (GST), vacuolar sorting receptors (VSR), multi-antimicrobial extrusion protein (MATE) were found, which were thought to be involved in the transportation of flavonoids from cytosolic biosynthesis to their vacuolar accumulation (Petrussa et al., 2013). Notably, genes from the Brown module were significantly enriched in pathways related to secondary metabolites biosyntheses, such as flavonoid biosynthesis and phenylpropanoid biosynthesis (Supplementary Figure S3C). Interestingly, a large number of genes encoding key enzyme (flavonoids-related structural genes) had been identified in these pathways, including genes encoding phenylalanine ammonia-lyase (*PAL*), cinnamate 4-hydroxylase (*4CH*), chalcone synthase (*CHS*), chalcone isomerase (*CHI*), flavonol synthase (*FLS*), dihydroflavonol 4-reductase (*DFR*), anthocyanin synthase (*ANS*), anthocyanidin reductase (*ANR*), and UDP-glycosyltransferase (*UGT*).

### Construction of Flavonoid-Related Gene Regulation Network

**FIGURE 5:**
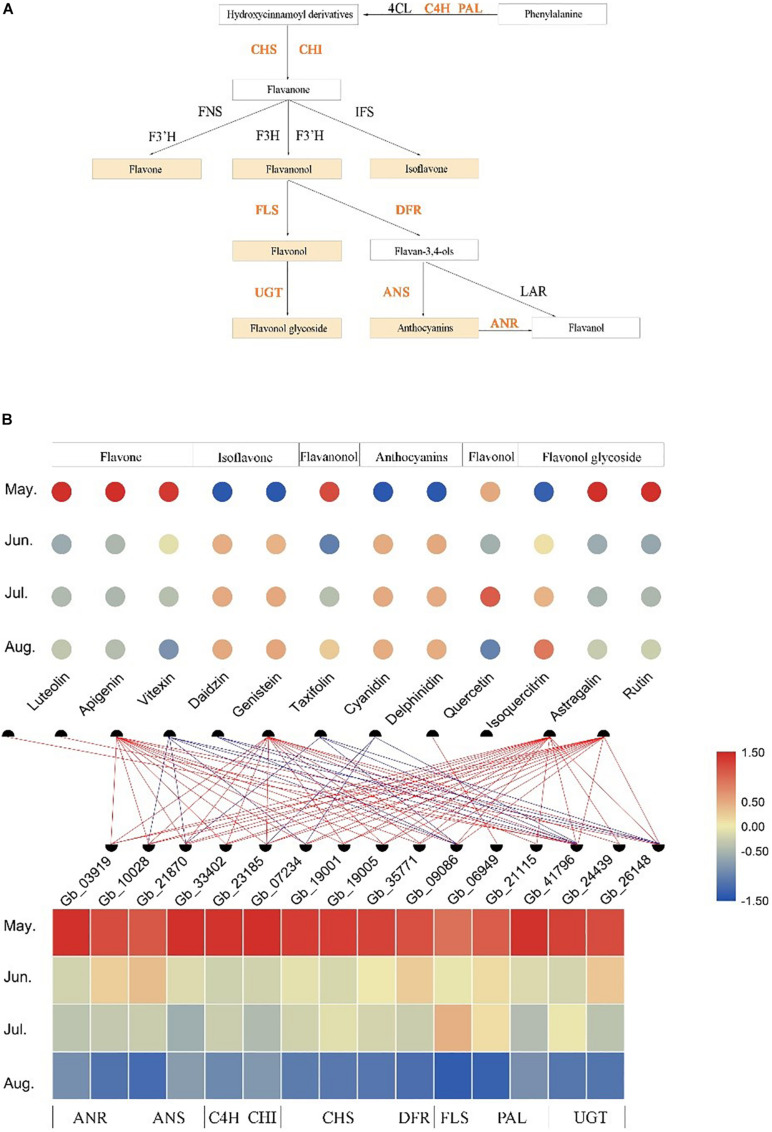
Temporospatial expression patterns of flavonoid-related structural genes in different leaf developmental stages (temporal) and association analysis with the accumulation of several kinds of flavonoids. **(A)** The biosynthesis pathways of several kinds of flavonoids. **(B)** The accumulation patterns of 12 flavonoids and the expression patterns of 15 flavonoid-related structural genes in different growth stages and the association analysis between them. The change in color of circle/rectangle from red to blue represents a gradual decrease in the abundance of flavonoids/expression of structural genes. The red line represents the positive correlation between the expression of structural genes and the abundance of flavonoids, and the blue line represents the negative correlation between them (*r* > | 0.6|, *P* < 0.05).

After screening the target modules, we constructed the biosynthesis pathways of six flavonoids (flavone, isoflavone, flavanonol, anthocyanins, flavonol, and flavonol glycoside) and identified the structural genes involved in these pathways from the Brown module (Figure 5A). The developmental stage specificity of the 12 flavonoids accumulation and the 15 structural genes expression was visualized (Figure 5B). We found that three flavones (luteolin, apigenin, and vitexin), one flavanonol (taxifolin), and two flavonol glycosides (astragalin and rutin) had the highest accumulation in May. In contrast, two isoflavones (daidzin and genistein), two anthocyanins (cyanidin and delphinidin), and one flavonol glycosides (isoquercitrin) had the lowest accumulation in leaves at the same developmental stage. Additionally, the accumulation of one flavonol (quercetin) was the highest in July (see Supplementary Table S3 for quantitative values of the 12 identified flavonoids). The 15 structural genes identified in the Brown module had similar developmental expression patterns, with high expression in May and low expression in August. Correlation analysis of transcriptome and metabolome indicated that some structural genes were significantly correlated with specific flavonoids (*r* > | 0.6|, *P* < 0.05). For example, quercetin content was positively correlated with the expression of a gene encoding *FLS* enzyme, while cyanidin content was negatively correlated with the expression of some genes encoding *ANS*, *DFR*, and *UGT* enzyme. Thus, these structural genes may play crucial roles in the accumulation of some flavonoids.

**FIGURE 6:**
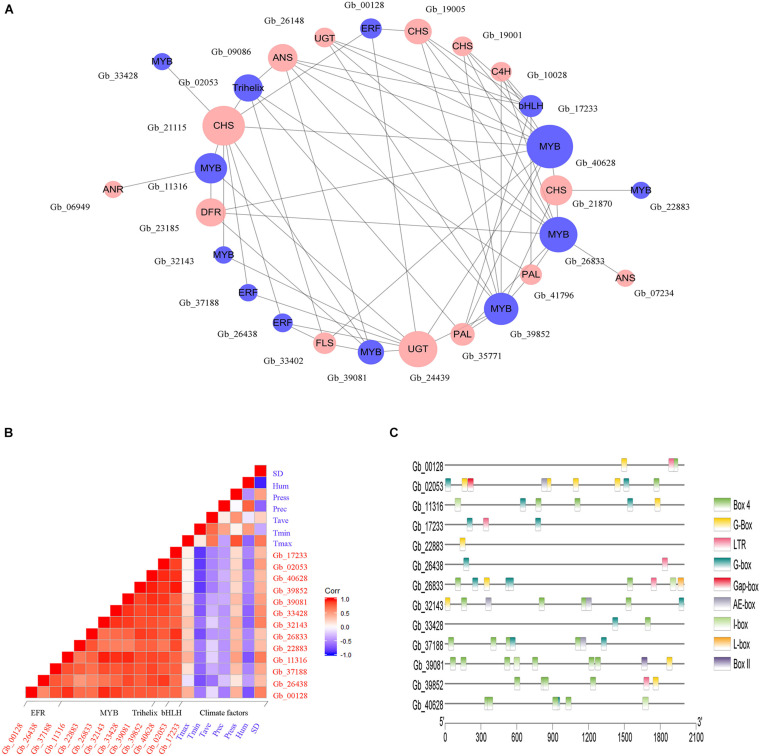
Environmental factors may trigger the activation of TFs to control the expression of flavonoid-related structural genes. **(A)** The co-expression network between TFs (blue circles) and flavonoid-related structural genes (red circles). The circle size is positively correlated with the connectivity of genes in the regulatory network. **(B)** The correlation analysis between environmental factors and the expression of TF genes. The red and blue square indicate positive and negative correlations between specific rows and columns, respectively. Climate factors include sunshine duration (SD), relative humidity (Hum), air pressure (Press), average temperature (Tave), minimum temperature (Tmin), and maximum temperature (Tmax). **(C)** *Cis*-regulatory elements analysis of TF genes.

In Brown module, a total of 13 genes belonging to four transcription factor families were identified, including those encoding *MYB* (8 genes), *ERF* (3 genes), *bHLH* (1 gene), and *Trihelix* (1 gene), which may be involved in the regulation of flavonoids accumulation. To explore the regulatory effect of TFs on flavonoid biosynthesis, a co-expression regulation sub-network was established among TF genes and flavonoid-related structural genes according to the correlation analysis (Figure 6A). We observed that *GbMYB* (Gb_40628) had the highest connectivity and was closely associated with 10 structural genes. Additionally, we observed that a structural gene was regulated by multiple TFs simultaneously, such as *GbCHI* (Gb_21115) was positively correlated with five *GbMYB*s (Gb_11316, Gb_32143, Gb_33428, Gb_39081, and Gb_40628), three *GbERF*s (Gb_00128, Gb_26438, and Gb_37188), and one *GbTrihelix* (Gb_02053). Therefore, we suggested that these TFs participated in the regulation of gene expression in the flavonoid biosynthesis pathway.

Further, the correlation between genes encoding TFs and climate factors was analyzed (Figure 6B). We found that several *GbMYB*s (Gb_11316, Gb_26833, Gb_32143, Gb_33428, and Gb_40628), *GbERF*s (Gb_26438 and Gb_37188), *GbbHLH* (Gb_17233), and *GbTrihelix* (Gb_02053) were significantly and negatively (*r* > | 0.6|, *P* < 0.05) correlated with mean minimum temperature (Tmin); three *GbMYB*s (Gb_11316, Gb_32143, and Gb_33428), one *GbERF* (Gb_00128), and one *GbTrihelix* (Gb_02053) had significant negative correlations with mean relative humidity (Hum); and two *GbMYB*s (Gb_11316 and Gb_32143) and one *GbERF* (Gb_00128) had significant positive correlations with sunshine duration (SD). These results suggested that the sunny environment was favorable to the expression of genes encoding TFs, while conversely the cold and humid environment was unfavorable to their expression. The promoter analysis also supported our hypothesis, as multiple light responsiveness (G-Box, Box 4, AE-box, I-box, L-box, Gap-box, Box II, and G-box) and low-temperature responsiveness (LTR) elements were found in the promoter regions of TF genes (Figure 6C).

## Discussion

Flavonoids represent one of the main classes of secondary metabolites that play an important role in plant defense against environmental stresses (e.g., temperature, precipitation, and light) (Akula and Ravishankar, 2011). Additionally, flavonoids extracted are also beneficial compounds for human health as cardioprotective, antihypertensive, and antioxidants (Fuchs et al., 2016). While studying the metabolic process of flavonoids in Ginkgo has been the subject of intense investigations (Wu et al., 2018; Meng et al., 2019; Guo et al., 2020), limited information is available at the genomic level. In the present study, we investigated the temporospatial (four leaves developmental stages and three contrasting test sites) transcriptome and metabolome dynamics biological processes to increase our understanding of Ginkgo’s flavonoids regulatory networks and to provide additional information of the molecular mechanisms of flavonoids accumulation during its leaves development. We used clonally propagated plant material, so the observed differences are attributable to either time (four leaves developmental stages) or space (three contrasting test sites).

### Temporospatial Gene Expression Profiles Variation

We used the RNA-seq approach to detect changes in the studied samples gene expression profiles and the principal component analysis, PCA-plot, clearly showed a spatial separation among samples growing at different environments, suggesting that gene expression at the transcriptome level is strongly influenced/modified by environmental conditions (Figure 2A). Observations confirming previously held view that transcriptional regulatory cascades may be key components of differential resilience shown by plants to changing environments (D’esposito et al., 2017). Furthermore, the four developmental stages also were separated (PCA-plot: Figure 2A), suggesting that the observed temporal differences provided a reasonable description of transcription activities during leaf development stages (Garg et al., 2017). Interestingly and more strikingly, the transcriptome from different spatial (environmental) samples showed similar stage-specific expression patterns that were gradually separated in the same direction (PCA-1) during leaf development. These findings reflected the dynamic nature and flexibility of gene expression in response to internal (genetic) and external (environmental) cues at the transcription level during leaf development (Bar and Ori, 2014).

### Flavonoids Metabolism Is Temporospatially Influenced

The observed differentially expressed genes (DEGs) among spatially different samples (environments) were identified and exhibited a collinear pattern with the environmental differences between sites. As the environmental differences between sites increase, this was accompanied by a concomitant increase in the differences of DFGs of their respective samples. For example, the difference between DEGs from YN and QJ sites was in line with the observed differences between these two sites environments (Figure 3A). Ginkgo may have developed a genetic control system as a survival strategy in response to different environments (Cho et al., 2018; Wang et al., 2020). Further, the eight temporal expression patterns (DEG sets) identified by STEM analysis (Figure 3B), contained genes significantly affected by environmental (spatial) and developmental (temporal) processes. The KEGG pathway enrichment analysis (Figure 3C) indicated that the expression of genes from Profile 0 presented a down-regulated trend with leaf development, and these genes were significantly enriched in both phenylpropanoid and flavonoid biosynthesis pathways (ko 00940 and ko 00941). These results indicated that a set of genes related to flavonoid biosyntheses, such as structural genes and TF genes, performed the stage-specific (temporally sequenced) function under external environmental stimuli. It has been reported that there is a rhythm, a time-distribution character, to the biosynthesis and metabolism of flavonoids in Ginkgo leaves (Cheng et al., 2012; Sati et al., 2013). Our results indicated that flavonoid-related metabolism was more active at the transcriptional level in the early stage of leaf development, consistent with previous studies (Yan et al., 2019; Zhu et al., 2020). These findings were also confirmed by the result of HPLC analysis; where we found that the content of TFG peaked in the early stage (Figure 1A). We also found that samples from YN and QJ sites had the greatest difference in TFG content (Figure 1B).

### A Regulated Transcriptional Network for Flavonoids Biosynthesis

Previous studies have focused on the identification of flavonoid-related structural genes in Ginkgo leaves, such as genes encoding *PAL*, *C4H*, *4CL*, *CHS*, *CHI*, and *F3H* in early flavonoid biosynthesis pathway, and genes encoding *DFR*, *ANS*, and *ANR* in downstream steps of the pathway (Li et al., 2018; Wu et al., 2018). In the present study, an intensive association network was observed between the expression of 15 selected structural genes and abundance of flavonoids (Figure 5B), suggesting that these structural genes (*GbANR*, *GbANS*, *GbC4H*, *GbCHI*, *GbCHS*, *GbDFR*, *GbFLS*, *GbPAL*, and *GbUGT*) may play crucial roles in the accumulation of specific flavonoids. More specifically, we identified one gene (Gb_41796) encoding *PAL*, which is an upstream key enzyme and rate limiting of the flavonoids biosynthesis pathway (Wang et al., 2014), whose expression was positively correlated with the abundance of three flavones (luteolin, apigenin, and vitexin), but negatively correlated with the two isoflavones (daidzin and genistein). Additionally, we found two genes encoding *UGT* (Gb_24439 and Gb_26148) whose expression was significantly and positively correlated with the abundance of flavonoid glycosides (astragalin), consistent with and supporting previous studies (Cui et al., 2016; Zhu et al., 2020). The accumulation of anthocyanins has been reported to be positively correlated with the expression of *GbDFR*s (Ni et al., 2020), while we found the gene encoding *DFR* (Gb_09086) was negatively associated with cyanidin accumulation.

Flavonoids biosynthesis is mainly regulated by transcription factors at the transcription level (Xu et al., 2014; Cao et al., 2020). We constructed a co-expression regulation network among TF genes and flavonoid-related structural genes to explore their regulatory relationship (Figure 6A). We discovered eight *GbMYB*s that positively regulated the expression of multiple structural genes in the flavonoid biosynthesis pathway. *MYB* TFs represent one of the largest families of a transcription factor in plants, involving in the regulation of different biological processes (Dubos et al., 2010). The large number of *GbMYB*s in the Ginkgo genome indicated that each of them may involve unique functions. Meng et al. (2019) found that the *GbMYB5* was involved in the positive regulation of flavonoid biosynthesis, while Xu et al. (2014) suggested that the *GbMYBF2* was responsible for repressing flavonoid biosynthesis. *MYB* and *bHLH* can act individually or in concert with other TFs to regulate a series of structural genes involved in flavonoid metabolism (Terrier et al., 2009; Carletti et al., 2013). Similarly, this co-expression network showed positive correlations between *GbERF*, *GbbHLH*, and *GbTrihelix* and certain structural genes associated with flavonoids.

TFs are considered as the major regulators of gene expression in response to environmental changes. *MYB*, *ERF*, and *bHLH* have been shown to play important roles in regulating environmental stress responses (Nakashima et al., 2009; Agarwal and Jha, 2010). In this study, we found that some *GbTF*s expression was negatively correlated with mean minimum temperature but positively correlated with sunshine duration (Figure 6B). Meanwhile, we also identified abundant light responsiveness elements and LTRs elements in *GbTF*s promoter regions (Figure 6C). It has been confirmed in previous studies, anthocyanins accumulation in *Pinus contorta* seedlings grown under short sunlight was significantly lower than those growing in the long sunlight area; long-term light irradiation (16 h) on leaves of *Ipomoea batatas* generated a dramatic increase in flavonoids content (Camm et al., 1993; Carvalho et al., 2010). As the amount of sunlight increases, there is a concomitant rise in temperature, and the composition of flavonoids in *Ribes nigrum* has been found to be positively correlated with temperature (Zheng et al., 2012). Our findings further support that proper control of gene expression by TFs was essential for the flavonoids biosynthesis, which played an important role in response to environmental changes (López-Maury et al., 2008).

## Conclusion

Our investigation of the temporospatial transcriptome and metabolome dynamics biological processes provided new insights into the biosynthesis of flavonoids in Ginkgo leaves. We indicated that flavonoids content varied greatly at different developmental stages (temporally) and in different growth environments (spatially). Therefore, the careful selection of planting region(s) and optimization of leaf harvesting time are expected to substantially improve the benefits of Ginkgo utilization as a non-timber forest product. We constructed a co-expression regulation network and identified 13 TF genes having crucial roles in controlling the transcriptomic regulation of flavonoids by activating the expression of multiple structural genes. These results provide candidate genes for future enhancement of flavonoids production by genetic strategies in Ginkgo. Furthermore, the large amount of data resources generated will serve as the foundation for a system biology approach to study the dynamics of leaf development and flavonoids accumulation in other plants.

## Data Availability Statement

The datasets presented in this study can be found in online repositories. The names of the repository/repositories and accession number(s) can be found below: https://www.ncbi.nlm.nih.gov/Traces/study/?acc=PRJNA657336, SUB7912734.

## Author Contributions

GW and YE-K conceived the study. YG collected the field samples, analyzed the data, and drafted the manuscript. GW, YE-K, TW, and F-FF modified the manuscript. All the authors have approved the manuscript.

## Conflict of Interest

The authors declare that the research was conducted in the absence of any commercial or financial relationships that could be construed as a potential conflict of interest.

## Footnotes

1 http://gigadb.org/dataset/100613

2 https://cran.r-project.org/package=pheatmap

3 http://bioinformatics.psb.ugent.be/webtools/plantcare/html/

4 http://data.cma.cn/

## Supplementary Material

Supplementary Figure 1 UHPLC chromatogram of standards and samples at different sampling stages. **(A)** Standards; **(B–E)** samples collected from the Yi Ning (YN) site in May, June, July, and August, respectively.

Supplementary Figure 2 The qRT-PCR analysis results of key genes involved in the flavonoid biosynthesis. The FPKM **(A)** and relative expression levels **(B)** were presented in bar plot with the error bar by calculating the mean and stand deviation (SD) of three independent replicates.

Supplementary Figure 3 Bubble maps show the top 20 significantly enriched KEGG pathways among the genes in three modules highly associated with flavonoid accumulation.

Supplementary Table 1 Summary of the sequencing quality of 36 RNA libraries of Ginkgo leaves.

Supplementary Table 2 Primers used for the qRT-PCR assay.

Supplementary Table 3 Summary of the quantitative values (peak area) of the identified 12 flavonoids.
