# Supplementary material for: Temporospatial Flavonoids Metabolism Variation in Ginkgo biloba Leaves
Source: Front Genet. 2020 Nov 27;11:589326. doi: 10.3389/fgene.2020.589326 (PMC7728922; doi:10.3389/fgene.2020.589326)
Supplement: Supplementary Figure 1 — UHPLC chromatogram of standards and samples at different sampling stages. (A) Standards; (B–E) samples collected from the Yi Ning (YN) site in May, June, July, and August, respectively. [file Data_Sheet_1.docx]

Supplementary Materials

Table S1. Summary of the sequencing quality of 36 RNA libraries of Ginkgo leaves.

| **Developmental stages** | **Site** | **Raw reads** | **Clean reads** | **Total mapped reads** | **Reads mapped to gene (%)** |
| --- | --- | --- | --- | --- | --- |
| May | YN | 74,127,299 | 74,113,089 | 68,363,308 | 95.39 |
|  | PZ | 63,807,071 | 63,796,109 | 54,683,934 | 94.86 |
|  | QJ | 70,779,141 | 70,764,891 | 64,698,212 | 95.24 |
| June | YN | 46,040,807 | 45,986,471 | 42,532,701 | 93.05 |
|  | PZ | 55,534,038 | 55,479,404 | 50,970,715 | 94.04 |
|  | QJ | 57,046,133 | 56,990,855 | 52,029,908 | 92.94 |
| July | YN | 47,868,903 | 47,784,032 | 36,442,828 | 93.09 |
|  | PZ | 49,943,441 | 49,881,090 | 44,722,020 | 91.63 |
|  | QJ | 47,598,010 | 47,547,525 | 43,290,652 | 92.05 |
| July | YN | 49,405,587 | 49,348,249 | 45,281,543 | 94.22 |
|  | PZ | 43,777,341 | 43,727,046 | 36,711,823 | 91.14 |
|  | QJ | 52,204,321 | 52,162,193 | 48,285,824 | 93.76 |

Table S2. Primers used for the qRT-PCR assay.

| Gene symbol |  | Primer sequence (5' to 3') |
| --- | --- | --- |
| Gb_11130 | Forward primer | CCCACTCAGACCCCAACATC |
|  | Reverse primer | ATCAGAGCACCTGGAACGCA |
| Gb_19800 | Forward primer | AGATGAAGAAACTCCACCAA |
|  | Reverse primer | GAGAGACAAAAGCACACTGA |
| Gb_00766 | Forward primer | CGGTGTTGGTTTTCGACGAG |
|  | Reverse primer | CAGACTGACGGACAGATGCG |
| Gb_37561 | Forward primer | TCAATTTGTCGTTGGGCGGG |
|  | Reverse primer | CGGATGCTGGTGCTGGTGGT |
| Gb_23185 | Forward primer | GGTGGAACAAACCCGAAGAG |
|  | Reverse primer | AAATGGGCAGAGCCAGAATA |
| Gb_01672 | Forward primer | AGCAAAGTAGCCGTTGGGTG |
|  | Reverse primer | GCGTGTTCTTCTGTGTGAGG |
| Gb_14057 | Forward primer | GAATCTCCTTCAAACACACA |
|  | Reverse primer | TTTACTTTCATAGGCTCCTC |
| Gb_26256 | Forward primer | TACATCGGCTCTTGGCTCGT |
|  | Reverse primer | GGTTTTGCCCTGGTTTTCTG |
| Gb_14030 | Forward primer | ATGCCATCTCTCCCTCGCTC |
|  | Reverse primer | TAAAACTCCCATCCTCACCA |
| Gb_12629 | Forward primer | ATCTCAACAAAAGAAAACGC |
|  | Reverse primer | AGAAATCTATGTCCGGAACC |

Table S3. Summary of the quantitative values (peak area) of the 12 identified flavonoids.

| Developmental stages | Site | Luteolin | Apigenin | Vitexin | Daidzin | Genistein | Taxifolin | Cyanidin | Delphinidin | Quercetin | Isoquercitrin | Astragalin | Rutin |
| --- | --- | --- | --- | --- | --- | --- | --- | --- | --- | --- | --- | --- | --- |
| May | YN | 1.95E+06 | 3.25E+06 | 1.60E+06 | 2.24E+05 | 4.18E+05 | 4.97E+06 | -^1^ | - | 3.75E+05 | 6.27E+04 | 1.03E+08 | 1.18E+08 |
|  | PZ | 3.71E+06 | 3.98E+06 | 1.31E+06 | 1.24E+05 | 3.90E+05 | 2.30E+06 | - | - | 2.23E+05 | 5.86E+04 | 7.33E+07 | 9.26E+07 |
|  | QJ | 4.91E+06 | 6.24E+06 | 1.11E+06 | 9.80E+04 | 4.18E+05 | 3.34E+06 | - | - | 1.31E+05 | 4.22E+04 | 5.87E+07 | 9.23E+07 |
| June | YN | 4.20E+04 | 3.33E+05 | 1.47E+05 | 1.40E+06 | 2.06E+06 | - | 2.14E+06 | 3.60E+05 | 2.29E+04 | 3.13E+05 | 2.39E+05 | 2.63E+03 |
|  | PZ | 1.93E+04 | 3.94E+05 | 3.09E+05 | 1.22E+06 | 3.40E+06 | 2.80E+03 | 1.30E+06 | 2.55E+05 | 4.22E+04 | 2.55E+05 | 1.27E+06 | 6.68E+03 |
|  | QJ | 5.93E+04 | 4.91E+05 | 1.84E+05 | 1.10E+06 | 2.54E+06 | - | 1.66E+06 | 4.36E+05 | 8.96E+04 | 1.43E+05 | 8.67E+05 | 2.88E+04 |
| July | YN | 9.20E+03 | 4.99E+05 | 6.08E+04 | 1.42E+06 | 3.57E+06 | 6.33E+03 | 1.86E+06 | 2.06E+05 | 1.69E+06 | 3.67E+05 | 4.90E+05 | 5.71E+03 |
|  | PZ | 7.23E+04 | 4.57E+05 | 2.50E+05 | 1.26E+06 | 3.24E+06 | 1.33E+04 | 1.98E+06 | 2.39E+05 | 1.81E+04 | 4.57E+05 | 1.53E+06 | 1.95E+04 |
|  | QJ | 8.75E+04 | 2.61E+05 | 1.16E+05 | 1.18E+06 | 1.93E+06 | 7.83E+03 | 1.51E+06 | 5.01E+05 | 1.77E+04 | 1.92E+05 | 8.34E+05 | 5.17E+04 |
| August | YN | 1.47E+04 | 3.37E+05 | 5.47E+04 | 1.49E+06 | 3.80E+06 | - | 1.58E+06 | 1.79E+05 | 1.40E+04 | 3.77E+05 | 1.16E+06 | 4.08E+03 |
|  | PZ | 1.36E+05 | 5.58E+05 | 1.42E+05 | 1.41E+06 | 2.70E+06 | 2.90E+05 | 1.51E+06 | 1.90E+05 | 4.41E+04 | 8.61E+05 | 2.21E+06 | 7.41E+04 |
|  | QJ | 7.12E+04 | 4.14E+05 | 4.69E+04 | 9.74E+05 | 2.37E+06 | 1.63E+04 | 1.41E+06 | 5.26E+05 | 2.38E+04 | 3.66E+05 | 1.42E+06 | 1.34E+05 |

^1^ not present.

Supplementary Figures

**
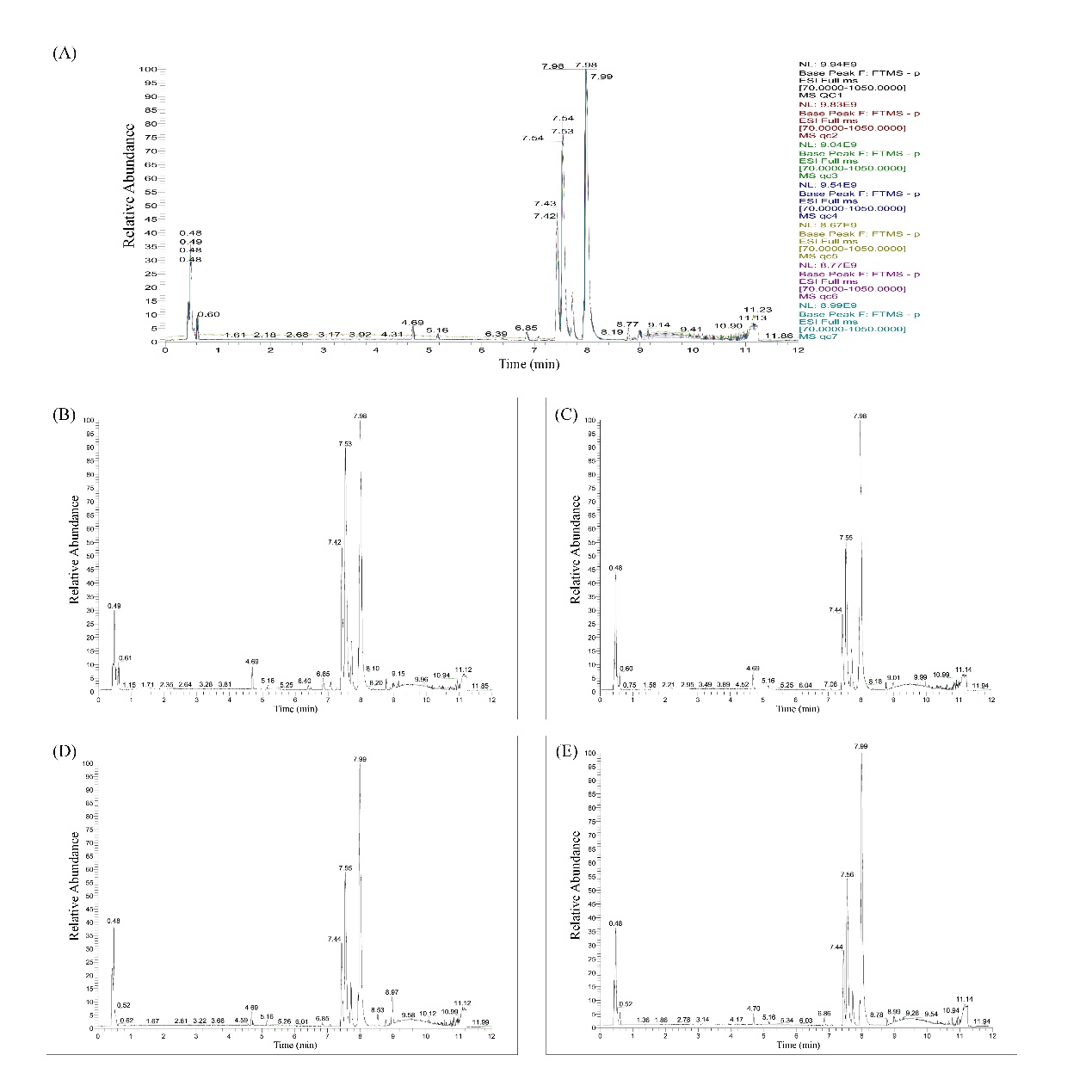
Figure S1.** UHPLC chromatogram of standards and samples at different sampling stages. (A) standards; (B, C, D, and E) samples collected from the Yi Ning (YN) site in May, June, July and August, respectively.


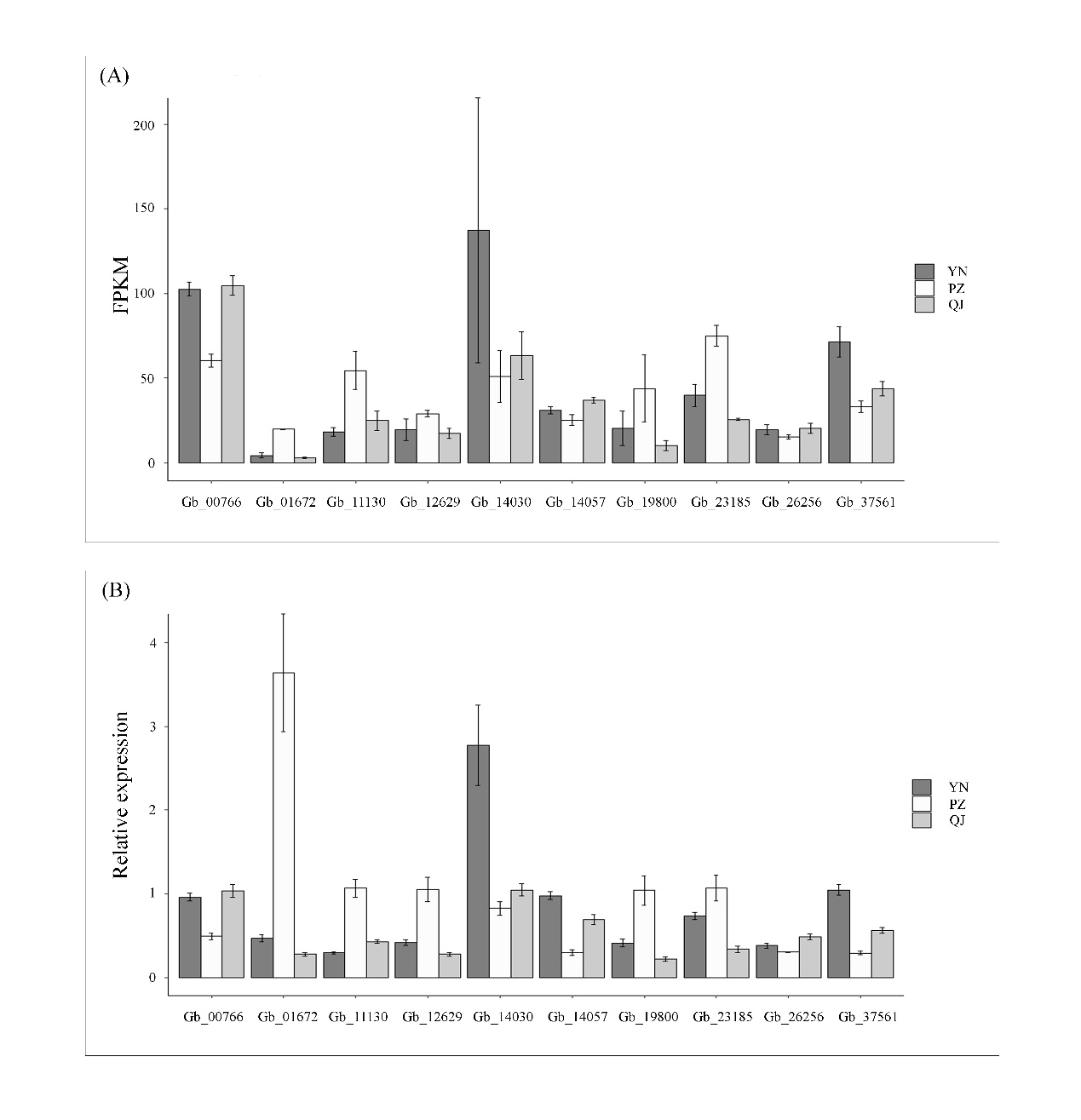


Figure S2. The qRT-PCR analysis results of key genes involved in the flavonoid biosynthesis. The FPKM (A) and relative expression levels (B) were presented in bar plot with the error bar by calculating the mean and stand deviation (SD) of three independent replicates.


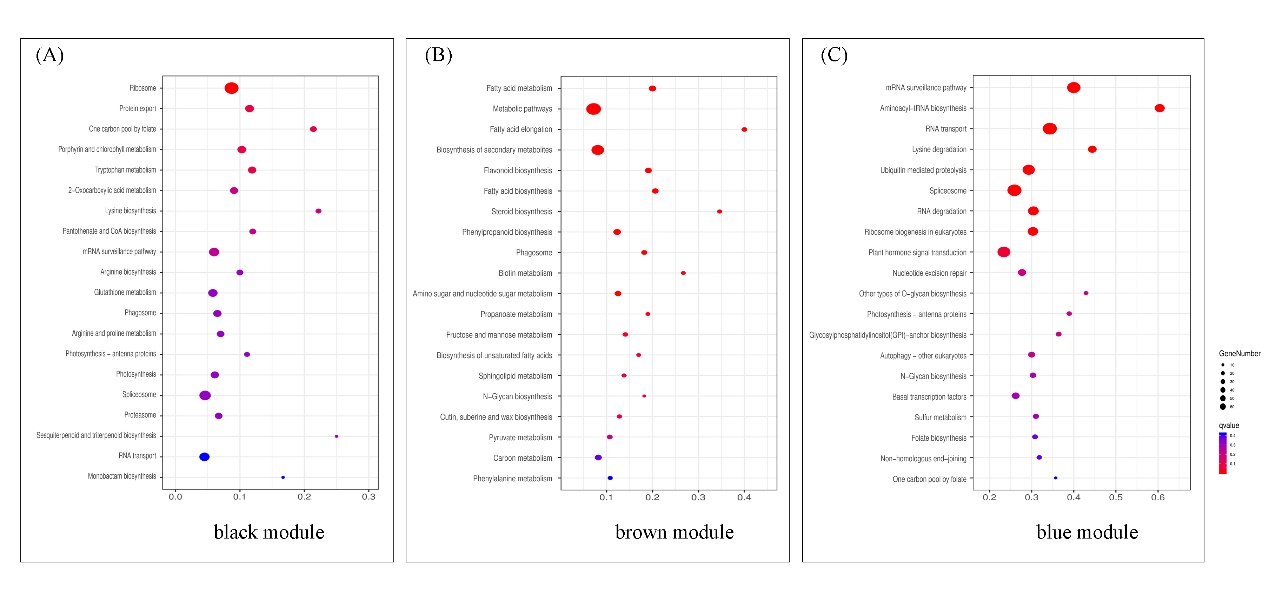


**Figure S3.** Bubble maps show the top 20 significantly enriched KEGG pathways among the genes in three modules highly associated with flavonoid accumulation.
